# Performance Evaluation of Commercial Dengue Diagnostic Tests for Early Detection of Dengue in Clinical Samples

**DOI:** 10.1155/2017/4687182

**Published:** 2017-12-12

**Authors:** Tuan Nur Akmalina Mat Jusoh, Rafidah Hanim Shueb

**Affiliations:** Department of Microbiology and Department of Medical Microbiology and Parasitology, School of Medical Sciences, Universiti Sains Malaysia, Health Campus, Kubang Kerian, Malaysia

## Abstract

The shattering rise in dengue virus infections globally has created a need for an accurate and validated rapid diagnostic test for this virus. Rapid diagnostic test (RDT) and reverse transcription-polymerase chain reaction (RT-PCR) diagnostic detection are useful tools for diagnosis of early dengue infection. We prospectively evaluated the diagnostic performance of nonstructural 1 (NS1) RDT and real-time RT-PCR diagnostic kits in 86 patient serum samples. Thirty-six samples were positive for dengue NS1 antigen while the remaining 50 were negative when tested with enzyme-linked immunosorbent assay (ELISA). Commercially available RDTs for NS1 detection, RTK ProDetect™, and SD Bioline showed high sensitivity of 94% and 89%, respectively, compared with ELISA. GenoAmp® Trioplex Real-Time RT-PCR and RealStar® Dengue RT-PCR tests presented a comparable kappa agreement with 0.722. The result obtained from GenoAmp® Real-Time RT-PCR Dengue test showed that 14 samples harbored dengue virus type 1 (DENV-1), 8 samples harbored DENV-2, 2 samples harbored DENV-3, and 1 sample harbored DENV-4. 1 sample had a double infection with DENV-1 and DENV-2. The NS1 RDTs and real-time RT-PCR tests were found to be a useful diagnostic for early and rapid diagnosis of acute dengue and an excellent surveillance tool in our battle against dengue.

## 1. Introduction

Dengue virus (DENV) infection is estimated to occur in over a billion throughout the tropical and subtropical regions of the world annually. This infection is devastating and recent evidence suggests that the infection has increased at an alarming rate from 2.2 million in 2010 to 3.2 million in 2015 [1]. Infection with DENV results in dengue fever as well as severe dengue and approximately 500,000 people with severe dengue require hospitalization each year [2]. The true numbers of the dengue cases are probably far worse, since severe underreporting and misclassification of infection have been documented [3].

DENV is arthropod-borne and is transmitted to humans mainly by the bite of an infected mosquito [4]. The primary vector is* Aedes aegypti* mosquito but other species such as* Aedes albopictus* have the ability to transmit the virus as well [5]. Dengue vector density diverges according to rainfall. In Malaysia, dengue is an important public health concern. Dengue occurs throughout this country with peak transmission occurring during late monsoon seasons. In 2017, the Malaysia Ministry of Health has reported 48,000 dengue cases in six months [6]. Dengue can cause as much or greater human suffering than other communicable diseases in some of the most affected regions [7]. Unfortunately, there is no specific antiviral medication to treat this viral infection. Hence, an accurate and early diagnosis is crucial for appropriate and prompt management and control of the disease in endemic regions [8].

Several diagnostic tools are available for detection of acute dengue infection, including virus isolation, viral nucleic acid detection, and viral NS1 antigen detection [9]. Lateral flow immunochromatographic assays or also known as RDTs are widely used for DENV NS1 antigen detection in many public health services as this antigen is detectable in patient sera as early as day one of fever. These RDTs take only about 15–20 minutes to get a result, are easy to perform, have low comparative cost when adopted for mass-surveys, and are convenient for distribution to clinical facilities set far away from main healthcare centers. Real-time RT-PCR (qRT-PCR) assay for dengue diagnosis has become increasingly available over the last five years because of the need for molecular diagnosis and dengue serotyping [10]. The qRT-PCR has been used for sensitive and specific detection of DENV RNA and hence diagnosis of dengue during acute phase of infection. However, unlike virus isolation, qRT-PCR can be completed in as quickly as two hours. In this study, the performance of two commercially available RDTs and three qRT-PCR kits for dengue virus was evaluated using serum samples from patients in an endemic area.

## 2. Material and Methods

### 2.1. Clinical Specimens

A total of 86 serum samples were used in this evaluation which include 81 samples from dengue suspected patients and five negatives specimens obtained from normal, healthy persons living in dengue nonendemic areas and were negative for DENV by RT-PCR and/or virus isolation. All these clinical samples were received and tested by the Virology Unit, Institute for Medical Research (IMR), Kuala Lumpur, and Department of Medical Microbiology & Parasitology, Universiti Sains Malaysia (USM), Kelantan, Malaysia (Ethical approval USM/JePeM/15110488).

### 2.2. Samples Preparation

All 86 samples were screened by enzyme-linked immunosorbent assay (ELISA) NS1 antigen assay prior to qRT-PCR. Detection of NS1 antigen from serum specimens was performed using the Platelia™ Dengue NS1 antigen capture test (Bio-Rad Laboratories, France) as the reference method as ELISA has better sensitivity and specificity than rapid tests. The testing was performed in strict adherence to the manufacturer's instructions provided in the kit. Briefly, the serum-MAb complex was added to 96-well microtiter plates precoated with a capture anti-NS1 MAb and the plates were then incubated at 37°C for 90 min. Subsequently, the complexes were detected by addition of 3,3′,5,5′-tetramethylbenzidine (TMB) substrate, and the reaction was stopped with 1 N sulphuric acid solution. Optical densities (OD) were determined within 30 min at 450/630 nm using a plate reader. The sample result was expressed by ratio which was determined by dividing the sample OD by the average for the cut-off control. NS1 ELISA positive samples were considered as dengue positive.

For qRT-PCR, RNA was extracted from 150 *μ*L of stored sample aliquots using Analytik Jena innuPREP Virus RNA kit, following the manufacturer's specifications. In brief, lysis solution was added to 150 *μ*L serum with subsequent binding of viral RNA onto surface of a spin filter membrane. The viral RNA was eluted from the membrane after several washing by using RNAse-free water. The extracted RNA was stored at −80°C until further use.

### 2.3. Diagnostic Kits Evaluation

#### 2.3.1. Evaluation of RDTs

For the evaluation performances of RDT, two lateral flow immunochromatographic tests for NS1 antigen detection, ProDetect Dengue Duo NS1 Antigen IgG/IgM rapid test (Mediven) and SD BIOLINE Dengue Duo® rapid test, were tested. All tests and result interpretation were performed in accordance with the manufacturer's instruction. The samples from patients presenting positive and negative results in NS1 ELISA were used for evaluating the performance of the two RDT tests. Sensitivity and specificity were assessed against reference test by ELISA.

#### 2.3.2. Evaluation of qRT-PCR

Three qRT-PCRs were evaluated in this study, RealStar Dengue RT-PCR, GenoAmp Trioplex Real-Time RT-PCR Zika/Den/Chiku, and GenoAmp Real-Time RT-PCR Dengue.

#### 2.3.3. RealStar Dengue RT-PCR (Altona Diagnostics, Hamburg, Germany)

The detection of the DENV was carried out using probes labeled with FAM fluorophore and BHQ quencher. An IC was provided and added to the specimen to detect inhibitors in the extraction. The testing was performed in strict adherence to the manufacturer's instructions provided in the kit. Samples and reagents were thawed prior to testing.

#### 2.3.4. GenoAmp Trioplex Real-Time RT-PCR Zika/Den/Chiku

The assay was based on the screening and differentiation of zika, pan-dengue, and chikungunya virus from clinical samples. DENV-specific target and target-specific probes were labeled with Texas Red reporter and quencher dyes. The target-specific probes for Zika and Chikungunya virus were labeled with FAM and HEX fluorophore, respectively. The assay consisted of an IC with the Cy5 fluorescence which was able to identify possible RT-PCR inhibition. Dengue primers and probes were designed to detect all DENV but could not differentiate their serotypes.

#### 2.3.5. GenoAmp Real-Time RT-PCR Dengue (Serotyping of Dengue 1–Dengue 4)

The serotyping of the positive samples was carried out using commercially available GenoAmp Real-Time RT-PCR Dengue kit. The assay contained a super mix for the specific amplification of DENV 1–4 RNA. The primer mix contained primers for DENV-1 carrying a Cy5 fluorophore, DENV-2 with the FAM fluorophore, DENV-3 with Texas Red probe, and DENV-4 carrying Cy5.5 probe. The primer mix included a HEX-labeled probe to detect the RNA internal control (IC) used to monitor the extraction process and RT-PCR inhibition. A positive control was provided in the kit. The test was carried out according to the manufacturer's instruction.

### 2.4. Statistical Analysis

Analyses of sensitivity and specificity were carried out by GraphPad Prism 7. Kappa agreement was also calculated using online available software (https://graphpad.com/quickcalcs/kappa2/). Kappa is a measure of the degree of nonrandom agreement between observers or measurements of the same categorical variable. Agreement is considered as good if kappa is between 0.60 and 0.80 and very good if greater than 0.80.

## 3. Results

### 3.1. DENV NS1 Detection by ELISAs and Evaluation of NS1 RDTs

From 86 samples tested by the ELISA method (Platelia Dengue NS1 antigen), 36 samples were identified as NS1 antigen positive while the remaining were negative. Following this, the performance of ProDetect and SD BIOLINE Dengue RDT kits were compared with the reference NS1 ELISA. Both dengue NS1 kits had high sensitivity (Table 1). A total of 34 (94.4%) positive NS1 samples were detected by ProDetect Dengue Duo NS1 Ag &IgG/IgM rapid test. The test had identified 48 (96%) negative samples. This RDT had a very good agreement with the NS1 ELISA with kappa value of 0.904 (95% CI: 0813–0.996). For SD BIOLINE Dengue kit, the sensitivity and specificity for the test were 88.9% and 100%, respectively. The kappa agreement (Ka) for this test also showed a very good agreement with the NS1 ELISA with kappa value of 0.903 (95% CI: 0810–0.995). The kappa agreement of both SD BIOLINE and ProDetect Dengue was comparable and thus, both tests were considered to be very good.

### 3.2. DENV qRT-PCR

Three commercial qRT-PCR kits were compared with the NS1 ELISA assay. For this analysis, 31 NS1 positive samples and 5 negative samples were used. As shown in Table 2, all the qRT-PCR kits tested in this study had high sensitivity (90.3%, 90.3%, and 83.9%). In addition, all kits showed 100% specificity when tested with negative samples. RealStar Dengue RT-PCR and GenoAmp Trioplex Real-Time RT-PCR Zika/Den/Chiku tests had good agreement with NS1 ELISA with kappa value of 0.722 (95% CI: 0.432–1.000). The performance of GenoAmp Trioplex Real-Time RT-PCR Zika/Den/Chiku was comparable to the RealStar Dengue RT-PCR. The kappa agreement between GenoAmp Real-Time RT-PCR Dengue and NS1 ELISA was 0.591 (95% CI: 0287–0.895).

### 3.3. DENV Real-Time RT-PCR Serotyping

As shown in Table 3, of the 26 dengue positive samples that were detected by GenoAmp Real-Time RT-PCR Dengue, 14 samples had DENV-1 infection. Eight and two samples were infected with DENV-2 and DENV-3, respectively, and one sample had DENV4. One sample displayed a pattern compatible with a double infection with DENV-1 and DENV-2. The GenoAmp® Dengue kit was able to subtype the dengue virus types 1–4 from clinical specimens and correctly identified the negative samples from healthy individuals. DENV-1 was the most prevalent serotype corresponding to ~40% of real-time PCR positive samples.

## 4. Discussion

For clinical management of dengue virus, early diagnostic is crucial for monitoring patients and providing prompt supportive therapy for patients who may progress to more severe forms of the disease [11, 12]. The performances of methods available for dengue diagnosis mainly depend on the disease period. Dengue viral RNA and soluble NS1 antigens are detectable during the early acute phase of infection, and thus, the sensitivity of virus isolation, qRT-PCR, and NS1 detection is higher on the first few days of illness [10]. Dengue hemorrhagic syndrome manifests when fever reduces, which occurs early in the infection; thus an effective clinical approach would benefit from early diagnosis [13]. However, given that there are many manufacturers for dengue NS1 antigen and qRT-PCR, performance evaluation on these tests is needed to ensure that the right test with high sensitivity and specificity is used.

The present study aimed to evaluate the performance of two commercially available rapid tests for dengue NS1 detection and three qRT-PCR assays in patient serum samples. This analytical study used specimens from laboratory confirmed dengue patients. The rapid tests and qRT-PCR had high sensitivity for dengue diagnosis. Both tests correlated well with serological diagnosis for case definition (positive NS1 ELISA) and the overall performance of the method was satisfactory when compared with NS1 ELISA.

DENV qRT-PCR kit was found to be practical and adjustable for high throughput. The design of various commercial molecular diagnostics kits vary with respect to the type of sample required (blood, plasma, or serum) and additional reagents [14]. Data from this study showed that all the tested qRT-PCR kits presented good sensitivity and specificity for dengue diagnosis. The tests could be performed with blood, plasma, and serum samples, while requiring only a comparable extraction kit for sample extraction. GenoAmp Real-Time RT-PCR Dengue kit is designed for the screening and differentiation of dengue virus serotypes (DENV-1, DENV-2, DENV-3, and DENV-4) in human plasma and serum, which provides an “add-on” value for patient management and for public health. A research by Fried et al. [15] showed that DENV-1 was the most commonly found serotype in study subjects, followed by DENV-3, DENV-2, and DENV-4. Since it was difficult to obtain a positive clinical sample with DENV-4, hence, only one DENV-4 sample was detected from the samples. The serotypes identified are consistent with the surveillance of dengue virus in Malaysia, whereby the predominance of dengue cases was attributable to DENV-1 [16]. This dominance of DENV-1 was preceded in time followed by DENV-2 and occasional DENV-3 cases [17, 18], so it is reasonable that only a few cases of these two serotypes were detected in this study.

Overall, our aim was to perform a comprehensive evaluation of methods for dengue diagnosis in patients during an epidemic period. In conclusion, the serological and real-time PCR tests for clinical use showed good diagnostic performance. All tests for dengue diagnosis have good performance of over 80% in detecting this disease and the test kits were easy to use and were very useful tools for the diagnosis of dengue in the early symptomatic phase.

## Figures and Tables

**Table 1:** Sensitivity, specificity, and kappa agreement of commercial RDTs for dengue NS1 detection in patient sera.

	Rapid diagnostic test	
	ProDetect	SD BIOLINE
Platelia NS1 (ELISA)		
Sensitivity (*n* = 36)	34 (94.4%)	32 (88.9%)
Specificity (*n* = 50)	48 (96.0%)	50 (100.0%)
Kappa agreement	0.904	0.903

**Table 2:** Sensitivity, specificity, and kappa agreement of qRT-PCR tests for detection of DENV in patient sera.

	qRT-PCR test		
	RealStar Dengue	GenoAmp Trioplex	GenoAmp Dengue
Platelia NS1 ELISA			
Sensitivity (*n* = 31)	28 (90.3%)	28 (90.3%)	26 (83.9%)
Specificity (*n* = 5)	5 (100.0%)	5 (100.0%)	5 (100.0%)
Kappa agreement	0.722	0.722	0.591

**Table 3:** Dengue serotypes identified by multiplex PCR in serum samples.

GenoAmp Real-Time RT-PCR Dengue	qRT-PCR	
Serotype	Positive	Percentage (%)
DENV-1	14	53.8
DENV-2	8	30.8
DENV-3	2	7.7
DENV-4	1	3.8
DENV-1 and DENV-2	1	3.8

Total	26	100
